# Presence of the apolipoprotein E-ε4 allele is associated with an increased risk of sepsis progression

**DOI:** 10.1038/s41598-020-72616-0

**Published:** 2020-09-25

**Authors:** Yiming Shao, Tian Zhao, Wenying Zhang, Junbing He, Furong Lu, Yujie Cai, Zhipeng Lai, Ning Wei, Chunmei Liang, Lizhen Liu, Yuan Hong, Xiaohong Cheng, Jia Li, Pei Tang, Weihao Fan, Mingqian Ou, Jingqi Yang, Yansong Liu, Lili Cui

**Affiliations:** 1 Guangdong Key Laboratory of Age-Related Cardiac and Cerebral Diseases, Affiliated Hospital of Guangdong Medical University, Zhanjiang, 524000 China; 2 The Intensive Care Unit, Guangdong Key Laboratory of Age-Related Cardiac and Cerebral Diseases, The Second Affiliated Hospital of Guangdong Medical University, Zhanjiang, 524000 China; 3 School of Clinical Medicine, Hubei University of Science and Technology, Xianning, 437000 China; 4 Zhanjiang Key Laboratory of Organ Injury and Protection and Translational Medicine, Guangdong, 524000 China; 5 The Intensive Care Unit, Jieyang Affiliated Hospital, Sun Yat-Sen University, Jieyang, 52200 China; 6 The Intensive Care Unit ,The Central Hospital of Wuhan, Tongji Medical College, Huazhong University of Science and Technology, Wuhan, 430000 China; 7 The Intensive Care Unit, The Second Affiliated Hospital of Harbin Medical University, Harbin, China

**Keywords:** Cell biology, Genetics, Diseases, Risk factors

## Abstract

Growing evidence indicated that single nucleotide polymorphisms (SNPs) in the apolipoprotein E (*APOE*) gene are related to increase the risk of many inflammatory-related diseases. However, few genetic studies have associated the *APOE* gene polymorphism with sepsis. This study was to investigate the clinical relevance of the *APOE* gene polymorphism in the onset and progression of sepsis. A multicenter case–control association study with a large sample size (601 septic patients and 699 healthy individuals) was conducted. Clinical data showed that the *APOEε4* allele was overrepresented among all patients with septic shock (*p* = 0.031) compared with sepsis subtype, suggesting that *APOEε4* allele may associated with increased susceptibility to the progression of sepsis. Moreover, the *APOE* mRNA levels decreased after lipopolysaccharide (LPS) stimulation in cells in culture. Then 21 healthy individuals to extract PBMC for genotype grouping (*APOE*4+ group 8; *APOE*4− group 13) was selected to evaluate the effect on APOE level, and results showed that the expression level of APOE in *APOE*4+ group and *APOE4*− *group* did not differ in mRNA levels after an LPS challenge, but the protein levels in *APOE4*+ group decreased slower than that in *APOE4*− *group*, and this process was accompanied by the upregulation of proinflammatory cytokines. These results provide evidence that the *APOEε4* allele might be associated with the development of sepsis and a potential risk factor that can be used in the prognosis of sepsis.

## Introduction

Sepsis due to infection is a complex disease that results in organ dysfunction, according to the latest definition for sepsis (Sepsis 3.0)^1^. Although progress in the development of antibiotics and other supportive care therapies, sepsis still causes at least one-third of hospital deaths^2,3^. The pathophysiological mechanisms that underlie sepsis are unclear, but growing evidence indicates that single nucleotide polymorphisms (SNPs) in genes play a significant role in the pathogenesis of sepsis and even contribute to sepsis susceptibility, progression, and prognosis^4–12^. Therefore, identifying genes that are associated with sepsis and evaluating their effects on gene expression and protein function can contribute to an increased understanding of the mechanism of sepsis occurrence and progression.

Apolipoprotein E (APOE) is a 34 kDa glycosylated protein, with relatively high amounts expressed in brain- and monocyte-derived macrophages^13^. In addition to its role in cholesterol transport and lipid metabolism, *APOE* has been shown exert immunomodulatory effects in vitro on both innate and acquired immune responses, as evidenced by its ability to suppress the proliferation of lymphocytes, the generation of cytolytic T-cells, and the stimulation of cultured neutrophils. These functions suggest that *APOE* has a potential role in various inflammatory-related diseases, including sepsis^14–18^. Increasing evidence has shown that *APOE* plays a critical role in the modulation of inflammatory processes by suppressing nuclear factor-κb-driven inflammation and atherosclerosis in monocytes and macrophages^19^. Recent studies have indicated that *APOE* knockout mice are highly susceptible to endotoxemia, *Listeria monocytogenes* and *Klebsiella pneumoniae* infection^20,21^. Rensen et al. showed that *APOE* redirect lipopolysaccharide (LPS) from Kupffer cells to hepatocytes and protect against endotoxemia in rats^22^. Other studies have demonstrated that the genomic deletion of *APOE* in mice resulted in an increased inflammatory reaction and high mortality rates following sepsis^23–26^. These lines of evidence suggest that *APOE* may have anti-infective and anti-inflammatory properties, which play a significant role in the pathogenesis of directly inflammatory-related diseases, such as sepsis.

The human *APOE* gene is located on chromosome 19q13.32 and exhibits polymorphism. There were three common *APOE* alleles, namely, ε2, ε3, and ε4, encoding the *APOE*2, *APOE*3, and *APOE*4 isoforms, respectively, resulting in six genotypes: *APOE*2/2, *APOE*2/3, *APOE*2/4, *APOE*3/3, *APOE*3/4, and *APOE*4/4^18,27–30^. Numerous of studies have shown that *APOE* gene polymorphisms result in genetic predisposition to various inflammation-related disease, such as Alzheimer’s disease^31^, coronary heart disease^32^, and multiple sclerosis^33^. However, the clinical relationship between *APOE* polymorphism and the development of sepsis is not well known, and clinical observations have been unsystematic.

The present study used multicenter data to investigate the association between *APOE* gene polymorphism and sepsis. In total, 601 septic patients and 699 healthy subjects from three regions—northern, central, and southern China—were included in the study to evaluate the clinical relevance of the *APOE polymorphism* in the susceptibility and progression of sepsis and explore the relationship between polymorphism in this gene and sepsis.

## Results

### Clinical characteristics of patients and healthy controls

**Figure 1:**
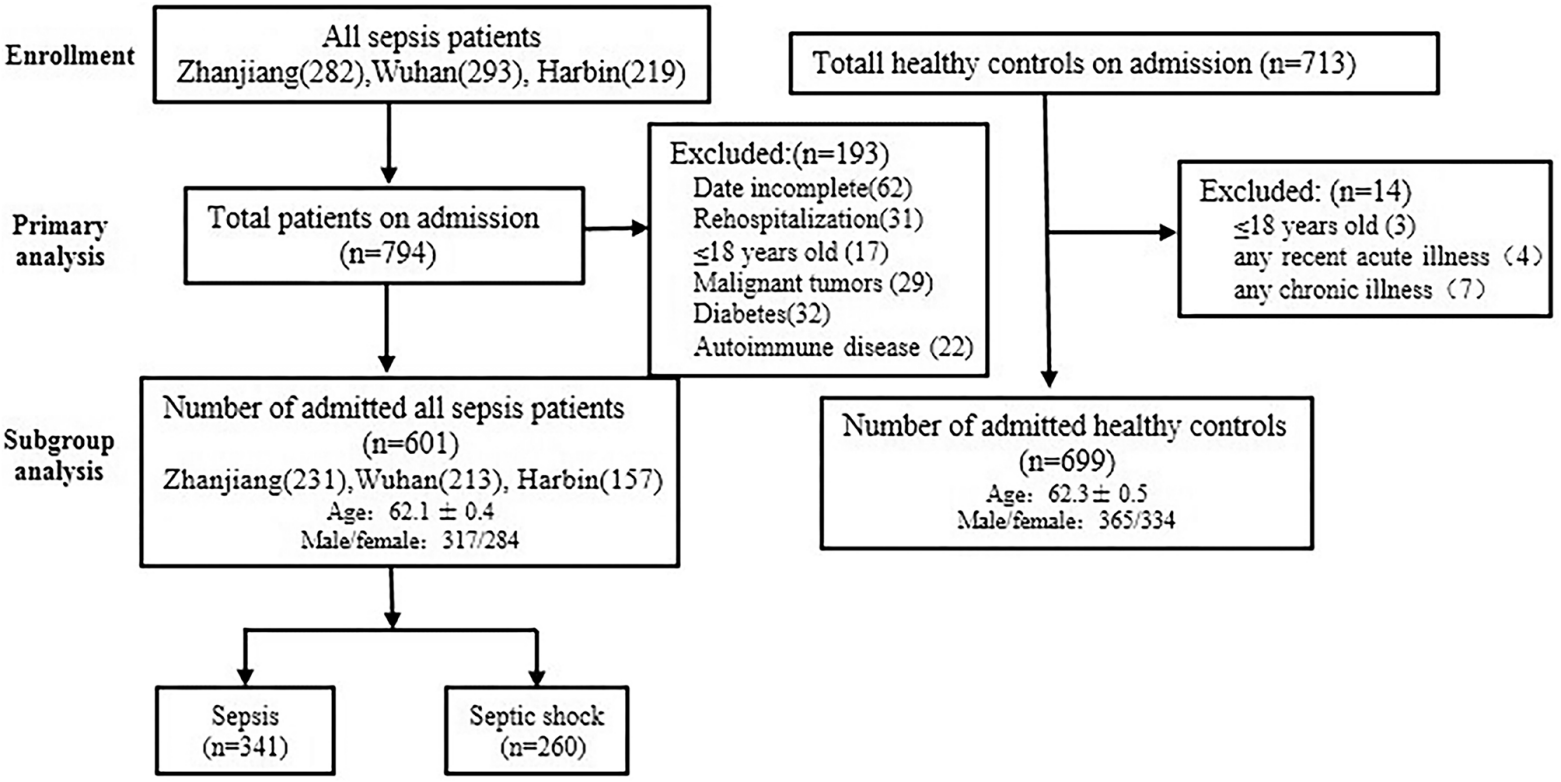
Flowchart of subject inclusion into clinical analysis cohort. Main procedures of clinical studies include sample collection, primary analysis and subgroup analysis. The participants included 282 sepsis patients from the Affiliated Hospital of Guangdong Medical College in southern China (Zhanjiang, China), 293 sepsis patients from the Center Hospital of Wuhan in central China (Wuhan, China) and 219 sepsis patients from Harbin Medical University in northern China (Harbin, China). Total healthy controls on admission is 713. In total, 601 sepsis patients and 699 healthy controls were included in the primary analysis.

In total, 699 healthy controls and 601 septic patients from three areas (Harbin, Wuhan, and Zhanjiang city) in China were enrolled in the study. The demographic characteristics of the 601 studied patients are shown in Table 1. The mean ages of the sepsis subtype and septic shock patients were 62.4 ± 0.3 years and 61.6 ± 0.4 years, respectively. There were no significant differences in age or sex distributions between the sepsis subtype and septic shock patients. The primary source of blood infection was lung infection. The most common type of infection was Gram-negative bacterial infections, which accounted for 33.4% of sepsis subtype cases and 34.2% of septic shock cases. The most common pathogens identified was *Acinetobacter baumannii*. The mean ages of the 699 healthy controls was 62.3 ± 0.5 years, including 365 male and 334 female. No significant differences in age and gender were observed between all sepsis patients and controls (Fig. 1).

**Table 1:** Baseline characteristics of sepsis cohort.

Variable	Total sepsis (n = 601) N (%)	Sepsis subtype (n = 341) N(%)	Septic shock (n = 260) N (%)
*Demographics*			
Age, mean ± SEM	62.1 ± 0.4	62.4 ± 0.3	61.6 ± 0.4
Male/female, number	317/284	180/161	137/123
*Source of infection, n (%)*			
Respiratory tract infection	338/601 (56.2)	194/341 (56.9)	144/260 (55.4)
Primary bloodstream infection	72/601 (12.0)	42/341 (12.3)	30/260 (11.5)
Abdominal infection	53/601 (8.8)	29/341 (8.5)	24/260 (9.2)
Urinary tract infection	49/601 (8.2)	27/341 (7.9)	22/260 (8.5)
Catheter-associated infection	11/601 (1.8)	7/341 (2.1)	4/260 (1.5)
Brain	26/601 (4.3)	14/341 (4.1)	12/260 (4.6)
Others	52/601 (8.7)	28/341 (8.2)	24/260 (9.2)
*Infection types, n (%)*			
Gram-positive	65/601 (10.8)	39/341 (11.4)	26/260 ( 10.0)
Gram-negative	203/601 (33.8)	114/341 (33.4)	89/260 (34.2)
Mixed gram-negative and -positive	64/601 (10.6)	38/341 (11.1)	26/260 (10.0)
Fungus	118/601 (19.6)	66/341 (19.4)	52/260 (20.0)
Polymicrobial	90/601 (15.0)	50/341 (14.7)	40/260 (15.4)
Negative blood culture	61/601 (10.1)	34/341 (10.0)	27/260 (10.4)
*Pathogenic bacteria, n (%)*			
*Acinetobacter baumannii*	140/601 (23.3)	78/341 (22.9)	62/260 (23.8)
*Monilia albican*	48/601 (8.0)	30/341 (8.8)	18/260 (6.9)
Yeast sample sporphyte	47/601 (7.8)	28/341 (8.2)	19/260 (7.3)
Aspergillus	43/601 (7.2)	26/341 (7.6)	17/260 (6.5)
*Klebsiella pneumoniae*	46/601 (7.7)	27/341 (7.9)	19/260 (7.3)
*Pseudomonas aeruginosa*	47/601 (7.8)	26/341 (7.6)	21/260 (8.1)
*Staphylococcus aureus*	49/601 (8.2)	22/341 (6.5)	27/260 (10.4)
*Escherichia coli*	67/601 (11.1)	41/341 (12.0)	26/260 (10.0)
Others	114/601 (19.0)	63/341 (18.5)	51/260 (19.6)
APACHE II score	24.09 ± 4.8	23.12 ± 4.2	26.46 ± 5.7

Clinical characteristics of sepsis and septic shock patients. APACHE II: Acute Physiology and Chronic Health Evaluation II; Continuous data are expressed as the mean ± SEM.

### The association between *APOE* gene polymorphism and sepsis susceptibility

The *APOE* genotype was successfully determined for all subjects. Genotypic distributions of *APOE* were consistent with Hardy–Weinberg equilibrium in the all sepsis patient and control groups (Table 2). The genotype distributions of *APOE* polymorphisms in the cases and controls of three groups are shown in Supplementary Table S1, the data shows that healthy volunteers and septic patients from three regions of Zhanjiang, Wuhan and Harbin have no statistically significant differences among the six genotypes of *APOE*. At the same time, we also analysis the genotype distributions of *APOE* polymorphisms in all patients and healthy controls in Table 3, the data shows that, there is also no differences among the six genotypes of *APOE* between patients and healthy controls. The patients were further separated into two subgroups according to genotype, regardless of clinical diagnosis: those possessing at least one *APOE*ε4 allele (2/4, 3/4, or 4/4, named the *APOE*4+ group) and those lacking *APOE*ε4 alleles (2/2, 2/3, or 3/3, named the *APOE4*− group)^34^. However, no significant differences in the *APOE* genotypes frequencies were observed between septic patients and healthy controls from Zhanjiang, Wuhan and Harbin in Supplementary Table S1 (all *p* > 0.05). However, Although not statistically significant, we observed that the frequency of carrying *APOE*ε4 allele in all patients with sepsis got higher trend than that of all healthy controls (Table 3) (*p* = 0.077).

**Table 2:** The Hardy–Weinberg equilibrium assay for *APO*E genotypes in all healthy controls, sepsis subtype, septic shock and all septic patients.

	*E*2/*E*2	*E*2/*E*3	*E*3/*E*3	*E*2/*E*4	*E*3/*E*4	*E*4/*E*4	*p*
Controls	7	100	482	11	93	6	0.997
Sepsis subtype	2	44	239	4	51	1	0.995
Septic shock	2	40	157	11	49	1	0.699
All	4	84	396	15	100	2	0.782

**Table 3:** *APOE* genotypic frequencies distribution in control and all sepsis patients.

Genotype	Control (n = 699)	All patients (n = 601)	*p* value	Chi square	Odds ratio (95% CI)
*APOE*4+	110 (15.7)	117 (19.5)	0.077	3.121	1.294 (0.972–1.724)
*APOE4*−	589 (84.3)	484 (80.5)			
*E*2/*E*2	7 (1.0)	4 (0.7)	0.510	0.435	0.662 (0.193–2.274)
*E*2/*E*3	100 (14.3)	84 (14.0)	0.865	0.029	0.973 (0.712–1.331)
*E*3/*E*3	482 (69.0)	396 (65.9)	0.239	1.385	0.870 (0.689–1.097)
*E*2/*E*4	11 (1.6)	15 (2.5)	0.236	1.402	1.601 (0.730–3.513)
*E*3/*E*4	93 (13.3)	100 (16.6)	0.092	2.842	1.301 (0.958–1.766)
*E*4/*E*4	6 (0.9)	2 (0.3)	0.227	1.460	0.386 (0.078–1.918)

*OR* odds ratio, *95% CI* 95% confidence interval ratio.

### The association between *APOE* gene polymorphism and sepsis progression

We further divided the septic patients into two subgroups—of sepsis subtype and septic shock—based on the severity of sepsis, to assess the effect of *APOE* SNPs on the progression of sepsis. As presented in the Supplementary Table S2, there is no differences between the genotypes of *APOE* in the sepsis subtype group and septic shock group from three regions. Next, we analyzed whether the *APOE* genotype is different between the total septic patients and septic shock patients. As shown in Table 4, the proportions of *E2*/*E2*, *E2*/*E3*, *E2*/*E4*, *E3*/*E4*, and *E4*/*E4* genotypes in all septic shock group were higher than those in the sepsis subtype group, with OR values of 1.314, 1.227, 3.722, 1.321 and 1.313 respectively, significantly higher frequency of *E*3/*E*3 genotype was observed in the sepsis subtype subgroup, *APOE*3/*E*3 genotype is protective compared to all other genotypes (OR = 0.65). Next, we divided all sepsis subtype subgroup and septic shock into two groups (*APOE*4+ and *APOE4*−) according to a specific allele. As presented in Table 4, in the *APOE*4+ group, more individuals had septic shock (*p* = 0.031, OR = 1.560) than sepsis subtype. We also divided all sepsis subtype subgroup and septic shock into *APOE*2+ and *APOE*2‒ or *APOE*3+ and *APOE*3‒ group in the Table 5, we found that in the *APOE*2+ group, there is a tendency to increase the development of sepsis (*p* = 0.066, OR = 1.490); In the *APOE*3+ group, lower individuals had septic shock (*p* = 0.028, OR = 0.368) than sepsis subtype. These findings suggest that carrying *APOE*ε4 allele may have a role in promoting the progression of sepsis from sepsis subtype to septic shock.

**Table 4:** *APOE* genotypic frequencies distribution in patients with sepsis subtype and septic shock.

Genotype	Sepsis subtype (n = 341)	Septic shock (n = 260)	*p* value	Chi square	Odds ratio (95% CI)
*APOE*4+	56 (16.4)	61 (23.5)	0.031	4.663	1.560 (1.040–2.340)
*APOE4*−	285 (83.6)	199 (76.5)			
*E*2/*E*2	2 (0.6)	2 (0.8)	1.000	0.000	1.314 (0.184–9.390)
*E*2/*E*3	44 (12.9)	40 (15.4)	0.385	0.755	1.227 (0.773–1.949)
*E*3/*E*3	239 (70.1)	157 (60.4)	0.013	6.180	0.651 (0.463–0.914)
*E*2/*E*4	4 (1.2)	11 (4.2)	0.017	5.668	3.722 (1.171–11.826)
*E*3/*E*4	51 (15.0)	49 (18.8)	0.205	1.610	1.321 (0.859–2.031)
*E*4/*E*4	1 (0.3)	1 (0.4)	1.000	0.000	1.313 (0.082–21.087)

*OR* odds ratio, *95% CI* 95% confidence interval ratio.

**Table 5:** The allele frequency distribution of *APOE*3+ and *APOE*3‒ groups or *APOE*2+ and *APOE*2‒ in all sepsis subtype subgroup and septic shock.

Genotype	Sepsis subtype	Septic shock	*p* value	Chi square	Odds ratio (95% CI)
	n = 341	n = 260			
*APOE*3+	334 (14.7)	246 (20.4)	0.028	4.856	0.368 (0.146–0.926)
*APOE*3‒	7 (85.3)	14 (79.6)			
	n = 341	n = 260			
*APOE*2+	50 (14.7)	53 (20.4)	0.066	3.401	1.490 (0.974–2.281)
*APOE*2‒	291 (85.3)	207 (79.6)			

### The association between *APOE* gene polymorphism and 30-day mortality in patients with sepsis genotypes

**Figure 2:**
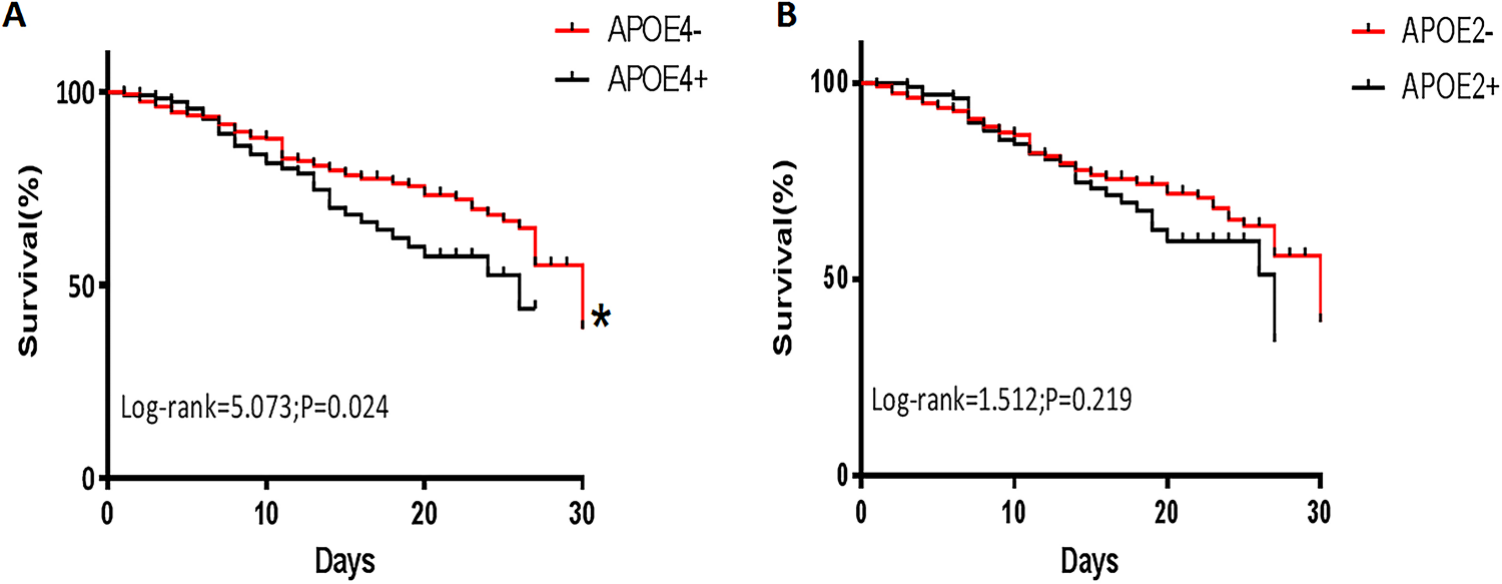
Kaplan–Meier survival analysis in all sepsis patients. The effect of *APOE*4+ and *APOE*4− genotype on the 30-day survival of all 601 patients from the Affiliated Hospital of Guangdong Medical University, the Center Hospital of Wuhan, and Harbin Medical University was assessed using Kaplan–Meier survival analysis (**A**); The effect of *APOE*2+ and *APOE*2− genotype on the 30-day survival of all 601 patients was assessed using Kaplan–Meier survival analysis (**B**).

The genotypic frequency distributions of the *APOE* in the two subgroups: *APOE*4+ and *APOE4*− groups or *APOE*2+ and *APOE*2‒ groups were stratified by 30-day mortality for further evaluation. Statistically significant difference was found between the 30-day surviving and non-surviving patients carrying the *APOE*4+ genotype (Supplementary Table S3, *p* = 0.041). Furthermore, Kaplan–Meier survival analysis showed that the 30-day survival of patients in the *APOE*4+ group (n = 117) was worse than that of patients in the *APOE4*− group (n = 484) (log-rank test 5.073, *p* = 0.024; Fig. 2A). Nevertheless, no difference was found between the 30-day surviving and non-surviving patients carrying the *APOE*2+ genotype (Supplementary Table S3, *p* = 0.053) and Kaplan–Meier survival analysis showed that no significant differences were observed in the *APOE*2+ and *APOE*2‒ group (log-rank test 1.512, *p* = 0.219; Fig. 2B).

### APOE is downregulated while expressions of inflammatory cytokines are elevated in sepsis patients and LPS-stimulated monocytes and macrophages in vitro

**Figure 3:**
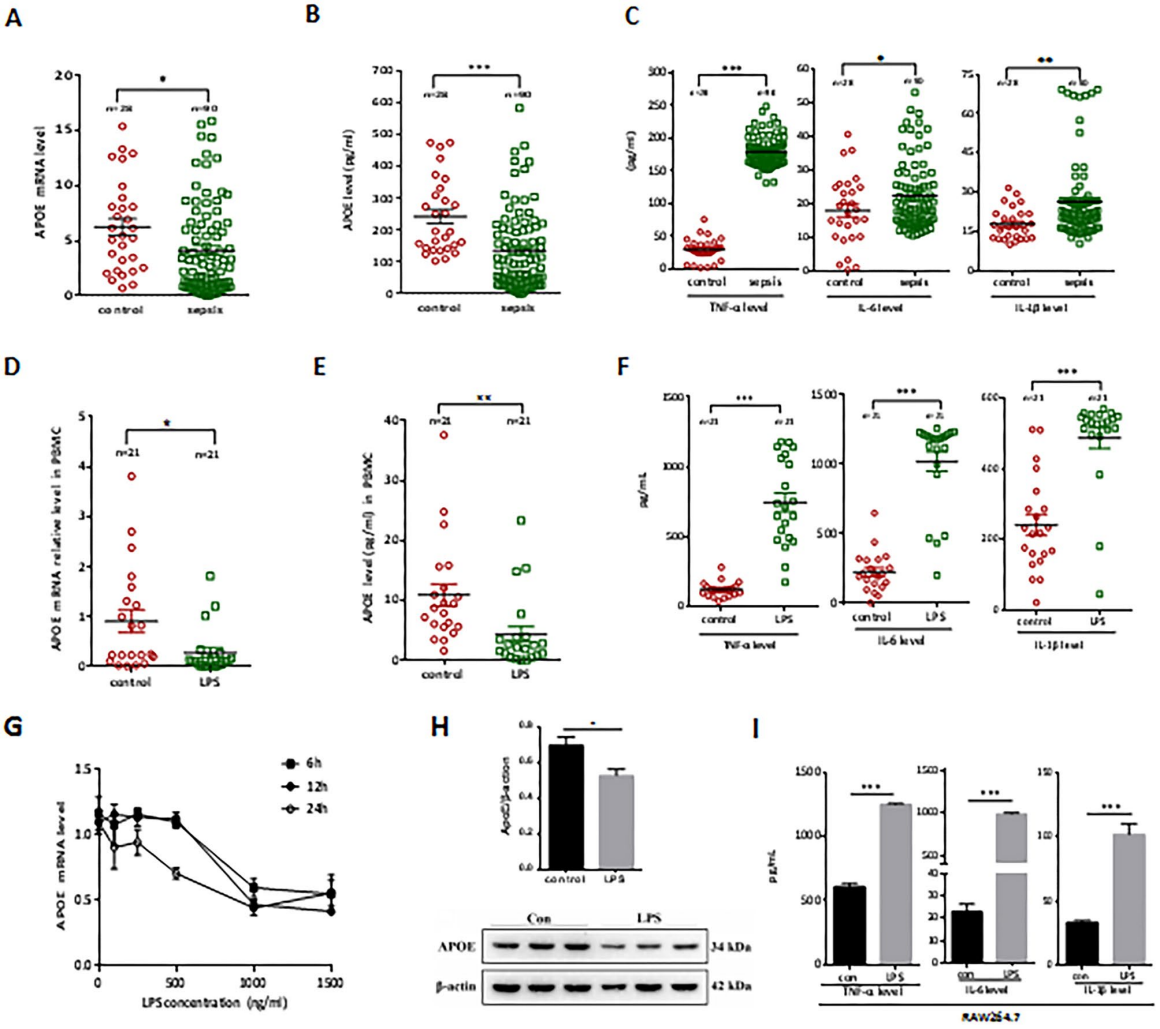
The APOE expressions and related inflammatory factors in sepsis patients and LPS-stimulated monocytes and macrophages in vitro. The *APOE* mRNA expression level, plasma concentration of APOE and related inflammatory factors in all sepsis patients (n = 90) and healthy controls (n = 28) (**A**–**C**); the *APOE* mRNA expression and the supernatant concentration of APOE in PBMCs from another 21 healthy individuals under 1000 ng/mL lipopolysaccharide (LPS) stimulation for 24 h (**D**,**E**); the related inflammatory factors of PBMCs under LPS (1000 ng/mL) stimulation for 6 h (**F**); dose responding viability of raw264.7 cells after 6, 12 and 24 h treatment with LPS (**G**);The effects of LPS on the expression of APOE in cultured raw264.7 cells for 24 h was detected by western blot analysis (**H**), because the strips were placed too close at the time, there was no long enough image, the original image is in the supplementary Figure S1; The related inflammatory factors of raw264.7 cells under LPS (1000 ng/mL) stimulation for 6 h (**I**); The error bar represents standard error of the mean. *p < 0.05; **p < 0.01; ***p < 0.001.

We randomly selected 90 septic patients and 28 healthy individuals to evaluate the plasma APOE level in vivo. The *APOE* mRNA and protein levels in septic patients were significantly lower than those of the healthy controls (Fig. 3A, B, *p* = 0.024 and *p* < 0.001, respectively), while the levels of TNF-α, IL-6, and IL-1β were elevated in sepsis patients (Fig. 3C). To assess the transcriptional and translational changes in APOE under the sepsis condition, we also measured *APOE* mRNA expression and protein production of PBMCS extracted from another 21 healthy controls under LPS stimulation. As shown in Fig. 3D–F, the mRNA expression and protein production of APOE were significantly decreased in LPS-stimulated PBMCs (*p* = 0.016 and *p* = 0.002, respectively), while the levels of TNF-α, IL-6, and IL-1β were elevated. Furthermore, RAW264.7 cells were challenged with LPS and the resulting mRNA and protein levels of APOE and expressions of inflammatory cytokines were detected. As shown by qPCR, the decrease in *APOE* after the LPS challenge occurred in a dose-dependent manner and reached a low point at 1000 ng/mL LPS. Thus, 1000 ng/mL was selected as the best LPS concentration for subsequent LPS challenge experiments using RAW264.7 cells (Fig. 3G). Next, we assessed the expression of the APOE protein in RAW264.7 cells after 1000 ng/mL LPS stimulation for 24 h. As shown in Fig. 3H, after LPS stimulation, the expression of the APOE protein decreased, while the levels of inflammatory factors TNF-α, IL-6, and IL-1β increased (*p* < 0.05) (Fig. 3I).

### The effect of APOE gene polymorphisms on the expression of APOE and related proinflammatory cytokines in LPS-stimulated PBMCs

**Figure 4:**
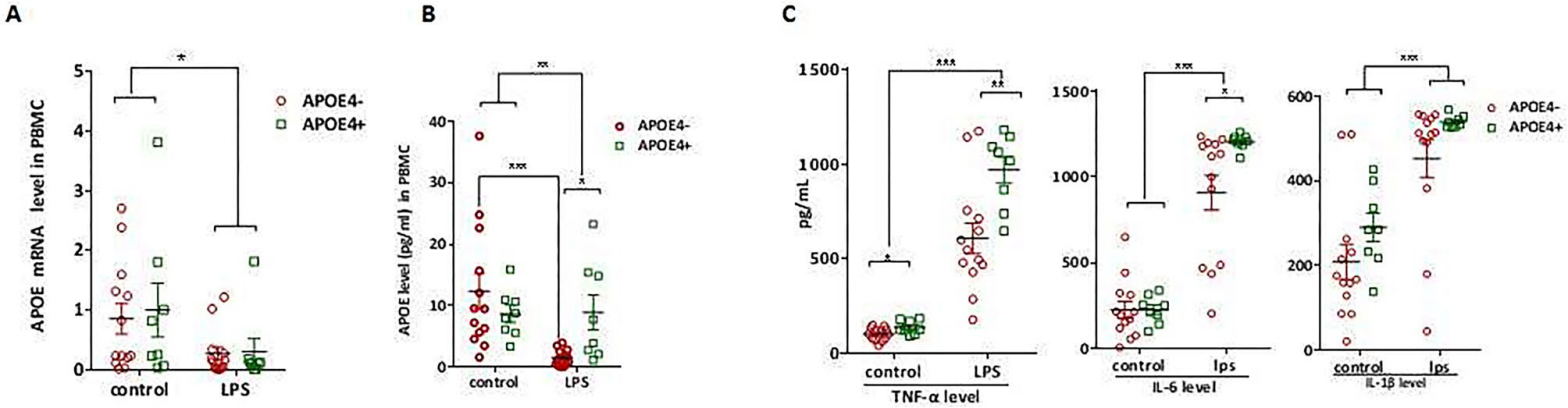
The APOE expression and related inflammatory factors with different APOE genotypes in LPS-stimulated monocytes in cells in culture. The *APOE* mRNA expression and the supernatant concentration in PBMCs from another 21 healthy individuals under 1000 ng/mL lipopolysaccharide (LPS) stimulation for 24 h in *APOE*4+ and *APOE*4− group (**A**,**B**); the related inflammatory factors in PBMCs under LPS (1000 ng/mL) stimulation for 6 h in *APOE*4+ and *APOE4*− group (**C**); the error bar represents standard error of the mean. *p < 0.05; **p < 0.01; ***p < 0.001.

Next we measured the mRNA and protein levels of APOE with different genotypes under the sepsis condition. An LPS challenge experiment was performed using PBMCs from the blood samples of healthy people, and the results are shown in Fig. 4. There was no difference in *APOE* mRNA levels among PBMCs with different genotypes, regardless of whether or not cells were stimulated by LPS (Fig. 4A, *p* > 0.05). After LPS stimulation, the supernatants APOE protein level in the *APOE4*− group was lower than that in the control (Fig. 4B, *p* < 0.001), whereas the protein level in the *APOE*4+ group showed no difference compared with the control (Fig. 4B, *p* > 0.05), however, the supernatants protein level in the *APOE*4+ group was higher than that in the *APOE4*− group (Fig. 4B, *p* < 0.01). To confirm the effect of different APOE genotypes on proinflammatory cytokines, we also measured the expression levels of TNF-α, IL-6, and IL-1β in LPS-stimulated PBMCs. In the supernatants of LPS-stimulated PBMCs isolated from 21 healthy volunteers with different APOE genotypes, the results showed that after LPS stimulation, the TNF-α and IL-6 levels in the *APOE*4+ group were higher than those in the *APOE4*− group (Fig. 4C, *p* = 0.006 and *p* = 0.037, respectively), while the IL-1β level did not differ between the *APOE*4+ and *APOE4*− groups (Fig. 4C, *p* > 0.05). These results indicated that *APOE*ε4 carriers may suffer a more excessive inflammatory response in sepsis.

## Discussion

Gene polymorphisms are critical for determining a predisposition to sepsis susceptibility and progression and could be used as potential therapeutic targets for the treatment of sepsis^8,9,35–40^. In vivo and in vitro studies have confirmed that APOE and *APOE* gene polymorphisms play a critical role in the modification of immune responses and inflammation, which, in turn, may contribute to the development of sepsis^14,18,20^. In this study, we investigated the association between sepsis and *APOE* in a multicenter case–control study. Our clinical data showed that patients in the *APOE*4+ group demonstrated a tendency toward an increased risk of sepsis, because the occurrence of sepsis is affected by many factors, whether the APOE gene polymorphism is related to the occurrence of sepsis needs to be further explored in a more uniform background and more samples, and a significant correlation was found between *APOE*4+ patients and increased sepsis mortality compared with *APOE4*−. Moreover, in the 30-day survival curve, significant differences in the *APOE* genotype were observed between surviving and non-surviving patients, with a worse outcome found for *APOEε*4 carriers compared with non-carriers. In addition, *APOE* mRNA and the plasma protein levels decreased under the sepsis condition. Further analysis revealed that the APOE protein level in the *APOE4*− group was lower than that in the *APOE*4+ group. This result is consistent with our clinical data, which indicate that the presence of the *APOEε4* allele may be a susceptibility gene for sepsis and a potential prognostic indicator.

APOE is a polymorphic protein that is involved in the transformation and metabolism of lipoproteins and the regulation of systemic inflammation^41^. *APOE* gene polymorphism is associated with many diseases, especially neurodegenerative diseases such as Alzheimer’s disease^42^. Previous studies have shown that patients with *APOE*3 deficiency are more likely to present with hyperlipidemia^43^. *APOE*3 protects porcine proximal tubular cells from gentamicin-induced injury^44^ and have protective effect in Alzheimer's disease^45^, this is consistent with our research results, which show that the proportion of *APOE E*3/E3 genotype in sepsis subtype is significantly higher than that in the septic shock group, suggesting that the *APOE E*3/*E*3 genotype is protective. Studies have also demonstrated that targeted replacement mice expressing human *APOE4* transgenes had increased plasma inflammatory factors, hepatic injury, and splenic lymphocyte apoptosis after a systemic lipopolysaccharide challenge^46,47^. The volunteers carrying the *APOE4* allele had higher plasma TNF-α and IL-6 levels than those carrying the *APOE3* allele after intravenous lipopolysaccharide administration^48^. These results suggest that the *APOEε4* allele may act as a pro-inflammatory factor. As a disease closely related to inflammation, sepsis was studied in association with the polymorphism of *APOE* gene in the present study, and our results suggest that the *APOE*ε4 allele contributed to aggravating the progression of sepsis from sepsis subtype to septic shock. Moreover, *APOE*ε4 carriers had a lower 30-day survival rate, suggesting that the *APOE*ε4 allele acted as a risk factor of mortality following sepsis.

Missense mutations at exons rs429358 and rs7412 underlie the three *APOE* allelic isoforms: ε2, ε3, and ε4^41^. The molecular basis of *APOE* polymorphism is cysteine–arginine interchanges at residues 112 and 158: *E*2 (Cys112–Cys158), *E*3 (Cys112–Arg158), and *E*4 (Arg112–Arg158)^49^. Previous studies have shown that the substitutions at positions 112 and 158 affect salt bridge formation within the protein, which ultimately affects receptor-binding activity and lipoprotein ‘preference’ for the APOE protein^49,50^. The different transportability of the APOE3 and APOE4 proteins with arginine affects the ability of macrophages to produce nitric oxide^34^. These findings support the view that the effects of the *APOE3* and *APOE4* genetic polymorphisms on disease risk are likely attributable to functional differences in the translated proteins. In this study, we confirmed that *APOE* mRNA levels were decreased in the blood during sepsis, which is consistent with the results of our in vitro experiments using LPS-stimulated PBMCs and RAW264.7 cells. This result is supported by a previous study that reported a decrease in *APOE* mRNA in 279 pediatric patients with bacterial infections^51^. We found that the APOE protein level was lower in patients compared with the controls; this is inconsistent with previous studies that reported increased plasma APOE protein levels in pediatric patients with bacterial infections compared with healthy controls^51^. It has been reported that the administration of exogenous APOE decreased mortality rates by downregulating the inflammatory cascade in *APOE*-deficient animals^23,46,52^, suggesting that the APOE protein has anti-inflammatory properties which supported our results.

After further analysis of the association between *APOE* typing and its expression level, we observed that the protein levels in *APOE*4+ and *APOE4*− group, rather than mRNA levels, changed in the cells stimulated by LPS in vitro. This result suggests that the effect of *APOE* gene polymorphism on sepsis risk is mainly caused by a variation in function of the protein expressed by different *APOE* genotypes rather than the regulation of *APOE* at the transcription level. At same time we found that the APOE protein level under LPS stimulation in the *APOE*4+ group was higher compared with that of no LPS stimulation in the *APOE4*− group. Furthermore, the presence of the *APOEε4* allele was associated with higher levels of the proinflammatory cytokines TNF-α and IL-6 in PBMCs stimulated by LPS. Considering that there are only a relatively small number of *APOE*4+ individuals in this part of the in vitro experiments, the results may be biased and large samples and multiple centers will be required for verification in the future.

Our results support that *APOE* gene polymorphism contributed to the prognosis and development of sepsis. Previous evidences suggest that the APOE intervention could relieve the excessive systemic immune response^6^. In this study we observed that the APOE protein seems to be consumed in the development of sepsis, the decreased mRNA level of *APOE* may lead to the decrease of protein level in pathological process of sepsis. Interestingly, we observed that APOE4 protein level seems to be consumed more slowly than APOE3 in serum of patients, associated with higher level of inflammatory cytokines and poor prognosis in clinical observation, this may be caused by the different function of APOE 3 and 4 protein. Meanwhile the mechanism on effect of APOE protein in sepsis is still unclear. It has been reported that Human APOE4 could modulate the expression of Sirtuin 1 in brain regions of targeted replacement apoE mice^53^. In addition, a small molecule *APOE4*− targeted therapeutic candidate that normalizes sirtuin 1 levels and improves cognition in an Alzheimer’s disease mouse model^54^, suggesting the potential APOE4 and Sirt1 pathways. It is worth nothing that Sirt1 was recently reported that playing critical role to preventing autoimmunity and participate in sepsis progress^55,56^. Sirt 1 is a nicotinamide adenine dinucleotide (NAD+) dependent class III histone deacetylase (HDAC) that targets nuclear factor kappa B (NF-kB), a critical transcription factor in the regulation of proinflammatory cytokine production to adapt gene expression to metabolic activity^57–59^. Moreover, SIRT1 could regulated immunometabolic polarity during the hyper-inflammatory and hypoinflammatory phases of sepsis^60^. The Sirt1 activator could also attenuate multiorgan injury in septic mice model, decrease the production of proinflammatory cytokines and reduce inflammasome activation, suggesting that Sirt1 may play an important role in sepsis through the NF-kb pathway^61^. So although the potential mechanism of APOE in sepsis is still unclear, these above evidence suggest that APOE4, Sirt1, and NF-kb pathways may be one of the critical pathways for the sepsis progress. Further study on the function of APOE4 may gradually clarify its molecular mechanism in sepsis.

## Conclusion

In the present study, we demonstrated that *APOE* polymorphism was associated with the progression of sepsis in a Chinese Han population. Individuals carrying the *APOE*ε4 allele exhibited an association with the progression of sepsis. A higher APOE4 protein level may contribute to the risk of transitioning from sepsis subtype to septic shock. Our future studies will focus on the mechanisms of APOE4 in the pathogenesis of sepsis and whether the *APOE*ε4 allele can be used as an early warning signals of genetics or the APOE protein could be as a therapy target for the treatment of sepsis in clinical practice.

## Methods

### Subject enrollment

All patients were from the general intensive care unit (ICU) of the Affiliated Hospital of Guangdong Medical University, the Center Hospital of Wuhan, and Harbin Medical University between August 2016 and December 2018. All of them were ethnic Han Chinese from different families and not blood-related. Patients were screened strictly according to The Third International Consensus Definitions for Sepsis and Septic Shock (2016), sepsis subtype was defined as life-threatening organ dysfunction caused by a dysregulated host response to infection, septic shock was defined as a subset of sepsis in which particularly profound circulatory, cellular, and metabolic abnormalities are associated with a greater risk of mortality than with sepsis alone^1^, and the final sample included 601 sepsis patients (mean age: 62.1 ± 0.4 years; 47.3% female) and 699 healthy controls (mean age: 62.3 ± 0.5 years; 47.8% female) from three region of China, respectively. The DNA of all subjects was obtained. Patients were not eligible if they were younger than 18 years old, diagnosed with HIV, known to be immunodeficient, taking steroids, receiving radiation therapy, pregnant, or lactating. Those failing to meet the definition and diagnostic criteria in Sepsis 3.0 were also excluded from the patient cohort. The participants in the healthy control group were excluded if they were less than 18 years old or suffered from chronic illness or any recent acute illness (Fig. 1)^10^. Once a diagnosis of sepsis was confirmed, peripheral blood samples were collected within 12 h. The baseline demographic factors and clinical information were recorded for each patient^62^.

### DNA extraction and genotyping

The TIANamp Blood DNA Kit (TianGen Biotech, Beijing, China) was used according to the manufacturer’s instructions to isolate the genomic DNA from whole blood samples. We used PCR-based restriction fragment length polymorphism (RFLP) analysis to identify the *APOE* genotype. Finally, we obtained E2/E2 (168 and 50 bp), E3/E3 (145, 50, and 23 bp), and E4/E4 fragments (195 and 23 bp). Different combinations defined the heterozygous genotypes^63,64^.

### Mononuclear cell isolation, plasma collection, and LPS stimulation

Sterile, preservative-free heparin (10 U/mL) was used in the collection of venous blood. Peripheral blood mononuclear cells (PBMCs) were recovered from the interface of a Lymphoprep density gradient, washed twice in RPMI-1640, and resuspended at a concentration of 1 × 10^6^ cells/mL. In vitro, PBMCs from 21 healthy volunteers, who were chosen from the healthy control group at random, were used for LPS stimulation experiments. As soon as possible, plasma was extracted from the blood samples by centrifugation at 3000×*g* for 10 min and stored at − 80 °C until they were used for cytokine measurements. The supernatants of cells stimulated by 1000 ng/mL LPS for 6 h were collected for the measurement of TNF-α, IL-6, and IL-1β. The supernatants and cells stimulated by 1000 ng/mL LPS for 24 h were used to detect *APOE* mRNA and protein levels. The control cells were treated with PBS instead of LPS.

### Cell culture

The mouse macrophage cell line RAW264.7 was obtained from the Shanghai Cell Bank. Cells were cultured in RPMI-1640 Medium (HyClone, Logan, Utah, USA) in 10% FBS in a humidified incubator containing 5% CO_2_ at 37 °C. The culture medium for PBMCs consisted of RPMI-1640 Medium (Thermo Fisher Scientific, Waltham, MA, USA) supplemented with 10% human serum, 20 mM HEPES (pH 7.3), 2 mM l-glutamine, 100 μg/mL streptomycin, and 100 U/mL penicillin. LPS (*Escherichia coli*, 055:B5, Sigma L-2880, Saint Louis, MO, USA) was reconstituted in PBS.

### RNA extraction, reverse transcription, and real-time PCR

RNA was extracted from PBMCs of the 21 healthy controls for in vitro LPS stimulation experiments by using the UNIQ-10 Column Trizol Total RNA Extraction Kit (Sangon Biotech, Shanghai, China). The RNA was reverse-transcribed using the First Strand cDNA Synthesis Kit (Thermo) following the manufacturer’s instructions. The integrity of the RNA was verified using 1% agarose gel electrophoresis. The expression levels of *APOE* were analyzed by quantitative real-time PCR with the SYBR Green method. The *APOE* primer sequences used in this assay were as follows: human 5′-GTTGCTGGTCACATTCCTGG-3′ (forward) and 5′-GCAGGTAATCCCAAAAGCGAC-3′ (reverse); mouse 5′-CTCCCAAGTCACACAAGAACTG-3′ (forward) and 5′-CCAGCTCCTTTTTGTAAGCCTTT-3′ (reverse). The GAPDH primer sequences used were as follows: human 5′-TGTGGGCATCAATGGAT-TTGG-3′ (forward) and 5′-ACACCATGTATTCCGGGTCAAT-3′ (reverse); mouse 5′-AAGAGGGATGCTGCCCTTAC-3′ (forward) and 5′-TACGGCCAAATCCGTTCACA-3′ (reverse). The relative level of each transcript was normalized to GAPDH. The relative expression levels of *APOE* mRNA from all participants were determined using the 2^−△△Ct^ method.

### Cytokine measurements

The concentrations of APOE, TNF-α, IL-6, and IL-1β in the supernatants of serum and the isolated PBMCs were measured using enzyme-linked immunosorbent assay (ELISA) kits according to the manufacturer’s instructions (Beyotime Biotechnology, Shanghai, China).

### Western blot analysis

Cells were harvested at the specified times and lysed with RIPA buffer (Beyotime, Shanghai, China) containing 2% PICT and 1% PMSF. The BCA protein assay kit (KeyGen Biotechnologies, Nanjing, China) was used to determine protein concentration. Then, 12% sodium dodecyl sulfate–polyacrylamide gel electrophoresis (SDS–PAGE) was used to separate the proteins. The membrane was blocked with 5% nonfat milk for 60 min at 37 °C and then incubated overnight with specific primary antibodies (anti-APOE, mouse monoclonal antibody, 1:1000 diluted, ab1906, Abcam, Cambridge, UK; anti-β-actin, 1:1000 diluted, Santa Cruz Biotechnology) at 4 °C, followed by horseradish peroxidase-conjugated goat anti-mouse IgG or goat anti-rat IgG secondary antibodies. The bands were analyzed using ImageJ software (National Institutes of Health, Bethesda, MD, USA) and normalized to β-actin.

### Statistical analyses

Statistical analysis was conducted using GraphPad Prism 6.0 (GraphPad Software Inc., San Diego, CA, USA) and SPSS version 19.0 (IBM, NY, USA). The association between *APOE* polymorphisms and sepsis was analyzed using the chi-squared test. The deviation of the allele or genotype frequency was analyzed using Hardy–Weinberg equilibrium (HWE). Student’s t-test and the Mann–Whitney U-test were used for normally distributed and non-parametric data, respectively. ANOVA was performed for all other calculations. The Kaplan–Meier method was used to plot 30-day survival curves for the different *APOE* genotypes, and the curves were compared using the log-rank test. Our data are expressed as the mean ± standard error of the mean (SEM) or as percentage frequencies. Statistical significance was defined as a *p* value < 0.05.

### Ethic declaration

This study was approved by the Ethics Committee of the Affiliated Hospital of Guangdong Medical University (Zhanjiang, China) and conducted according to the standards of the Declaration of Helsinki and written informed consents were obtained from all of the participants or their surrogates.

## Supplementary information

Supplementary Tables.

Supplementary Figure 1.

## Data Availability

The dataset used and analysed during the current study are available from the corresponding author on reasonable request.
