# Supplementary material for: Presence of the apolipoprotein E-ε4 allele is associated with an increased risk of sepsis progression
Source: Sci Rep. 2020 Sep 25;10:15735. doi: 10.1038/s41598-020-72616-0 (PMC7519096; doi:10.1038/s41598-020-72616-0)
Supplement: Supplementary file 1 — Supplementary Tables. [file 41598_2020_72616_MOESM1_ESM.pdf]

Title: Presence of the apolipoprotein E -ε4 allele is associated with an increased risk of sepsis progression

Yiming Shao[1,2,4+], Tian Zhao[1,3+], Wenying Zhang[1,3+], Junbing He[5], Furong Lu[3,5], Yujie Cai[1], Zhipeng Lai[3], Ning Wei[3], Chunmei Liang[1], Yuan Hong[3], Lizhen Liu[3], Xiaohong Cheng[3], Jia Li[6,], Pei Tang[1]，Weihao Fan[1]，Mingqian Ou[1]，Jingqi Yang[1]，Yansong Liu[7] and Lili Cui[1*+]

[1] Institute of Neurology, Guangdong Key Laboratory of Age-Related Cardiac and Cerebral Diseases, Affiliated Hospital of Guangdong Medical University, Zhanjiang, 524000, China

[2] The Intensive Care Unit, Guangdong Key Laboratory of Age-Related Cardiac and Cerebral Diseases, the Second Affiliated Hospital of Guangdong Medical University, Zhanjiang, 524000, China

[3] The Intensive Care Unit, Guangdong Key Laboratory of Age-Related Cardiac and Cerebral Diseases, Affiliated Hospital of Guangdong Medical University, Zhanjiang, 524000, China

[4] Zhanjiang Key Laboratory of Organ Injury and Protection and Translational Medicine, Guangdong, 524000, China

[5] The Intensive Care Unit, Jieyang Affiliated Hospital, Sun Yat-Sen University, Jeiyang, 52200, China

[6] The Intensive Care Unit ,The Central Hospital of Wuhan, Tongji Medical College, Huazhong University of Science and Technology. Wuhan, 430000, China

[7] The Intensive Care Unit, the Second Affiliated Hospital of Harbin Medical University, Harbin, China.

*Correspondence: cuilili@gdmu.edu.cn;

Table S1. *APOE* genotypic distribution from Zhanjiang, Wuhan and Harbin in Control and Sepsis patients.

Genotype	Control	All patients	p-value	Chi square	Odds ratio (95% CI)
**Zhanjiang**	n=366	n=231			
*E*2/*E*2	4(1.1)	2(0.9)	1.000	0.000	0.790（0.144-4.350）
*E*2/*E*3	56(15.3)	32(13.9)	0.627	0.236	0.890（0.557-1.423）
*E*3/*E*3	257(70.2)	162(70.1)	0.982	0.001	0.996（0.695-1.427）
*E*2/*E*4	4(1.1)	5(2.2)	0.483	0.492	2.002（0.532-7.535）
*E*3/*E*4	41(11.2)	28(12.1)	0.732	0.117	1.093（0.656-1.823）
*E*4/*E*4	4(1.1)	2(0.9)	1.000	0.000	0.790（0.144-4.350）
*APOE*4+	49(13.4)	35(15.2)	0.546	0.364	1.155（0.723-1.846）
*APOE*4-	317(86.6)	196(84.8)			
**Wuhan**	n=140	n=213			
*E*2/*E*2	1(0.7)	1(0.5)	0.767	0.088	0.656（0.041-10.569）
*E*2/*E*3	21(15.0)	24(11.3)	0.304	1.058	0.720（0.384-1.350）
*E*3/*E*3	88(62.9)	128(60.1)	0.602	0.272	0.890（0.574-1.380）
*E*2/*E*4	5(3.6)	7(3.3)	1.000	0.000	0.917（0.285-2.950）
*E*3/*E*4	24(17.1)	53(24.9)	0.085	2.967	1.601（0.935-2.742）
*E*4/*E*4	1(0.7)	0(0.0)	0.173	1.854	-
*APOE*4+	30(21.4)	60(28.2)	0.155	2.021	1.438（0.870-2.376）
*APOE*4-	110(78.6)	153(71.8)			
**Harbin**	n=193	n=157			
*E*2/*E*2	2(1.0)	1(0.6)	1.000	0.000	0.612（0.055-6.814）
*E*2/*E*3	23(11.9)	28(17.8)	0.119	2.435	1.604（0.883-2.915）
*E*3/*E*3	137(71.0)	106(67.5)	0.484	0.491	0.850（0.538-1.341）
*E*2/*E*4	2(1.0)	3(1.9)	0.816	0.054	1.860（0.307-11.274）
*E*3/*E*4	28(14.5)	19(12.1)	0.511	0.431	0.811（0.434-1.516）
*E*4/*E*4	1(0.5)	0(0.0)	0.275	1.193	-
*APOE*4+	31(16.1)	22(14.0)	0.595	0.283	0.852（0.471-1.540）
*APOE*4-	162(83.9)	135(86.0)			

OR：odds ratio, 95% CI ：95% confidence interval ratio

Table S2. *APOE* genotypic distribution from Zhanjiang, Wuhan and Harbin with Sepsis subtype and Septic shock.

Genotype	Sepsis subtype	Septic shock	p-value	Chi square	Odds ratio (95% CI)
**Zhanjiang**	n=110	n=121			
*E*2/*E*2	1(0.9)	1(0.8)	0.946	0.005	0.908（0.056-14.699）
*E*2/*E*3	15(13.6)	17(14.0)	0.928	0.008	1.035 (0.490-2.187)
*E*3/*E*3	82(74.5)	80(66.1)	0.162	1.955	0.666（0.377-1.179）
*E*2/*E*4	1(0.9)	4(3.3)	0.425	0.636	3.726（0.410-33.861）
*E*3/*E*4	10(9.1)	18(14.9)	0.178	1.810	1.748（0.769-3.970）
*E*4/*E*4	1(0.9)	1(0.8)	0.946	0.005	0.908（0.056-14.699）
*APOE*4+	12(10.9)	23(19.0)	0.086	2.940	1.917（0.904-4.066）
*APOE*4-	98(89.1)	98(81.0)			
**Wuhan**	n=126	n=87			
*E*2/*E*2	0(0.0)	1(1.1)	0.180	1.798	-
*E*2/*E*3	13(10.3)	11(12.6)	0.598	0.279	1.258（0.536-2.955）
*E*3/*E*3	81(64.3)	47(54.0)	0.133	2.260	0.653（0.374-1.140）
*E*2/*E*4	2(1.6)	5(5.7)	0.200	1.646	3.780（0.716-19.950）
*E*3/*E*4	30(23.8)	23(26.4)	0.663	0.190	1.150（0.613-2.156）
*E*4/*E*4	0(0.0)	0(0.0)	-	-	-
*APOE*4+	32(25.4)	28(32.2)	0.279	1.172	1.394（0.763-2.547）
*APOE*4-	94(74.6)	59(67.8)			
**Harbin**	n=105	n=52			
*E*2/*E*2	1(1.0)	0(0.0)	0.369	0.808	-
*E*2/*E*3	16(15.2)	12(23.1)	0.227	1.458	1.669（0.723-3.851）
*E*3/*E*3	76(72.4)	30(57.7)	0.064	3.421	0.520（0.259-1.045）
*E*2/*E*4	1(1.0)	2(3.8)	0.231	1.435	4.160（0.368-46.972）
*E*3/*E*4	11(10.5)	8(15.4)	0.375	0.788	1.554（0.584-4.134）
*E*4/*E*4	0(0.0)	0(0.0)	-	-	-
*APOE*4+	12(11.4)	10(19.2)	0.185	1.757	1.845（0.739-4.607）
*APOE*4-	93(88.6)	42(80.8)			

OR：odds ratio, 95% CI ：95% confidence interval ratio

Table S3. The allele frequency distribution in the *APOE*4+ and *APOE*4- groups or *APOE*2+ and *APOE*2- between 30-day surviving and non-surviving sepsis

Genotype	Survivors	Non-survivors	P
	n=464	n=137	
*APOE*4+	82	35	0.041
*APOE*4-	382	102	
	n=464	n=137	
*APOE*2+	72	31	0.053
*APOE*2-	392	106	
