# Supplementary material for: Presence of the apolipoprotein E-ε4 allele is associated with an increased risk of sepsis progression
Source: Sci Rep. 2020 Sep 25;10:15735. doi: 10.1038/s41598-020-72616-0 (PMC7519096; doi:10.1038/s41598-020-72616-0)
Supplement: Supplementary file 2 — Supplementary Figure 1. [file 41598_2020_72616_MOESM2_ESM.doc]

**Title: Presence of the** **apolipoprotein E -ε4 allele is associated with an increased risk of sepsis progression**

**Yiming Shao^1,2,4+^****, Tian Zhao^1,3+^, Wenying Zhang^1,3+^,** **Junbing He^5^, Furong Lu^2,5^,** **Yujie Cai^1^,** **Zhipeng Lai^3^,** **Ning Wei^3^,** **Chunmei Liang^1^,** **Yuan Hong^3^,** **Lizhen Liu^3^,** **Xiaohong Cheng^3^,** **Jia Li^6^, ,** **Pei Tang^1^，Weihao Fan^1^，Mingqian Ou^1^，Jingqi Yang^1^，****Yansong Liu^7^ and Lili Cui^1^*^+^**

^1^ Institute of Neurology, Guangdong Key Laboratory of Age-Related Cardiac and Cerebral Diseases, Affiliated Hospital of Guangdong Medical University, Zhanjiang, 524000, China

^2^ The Intensive Care Unit, Guangdong Key Laboratory of Age-Related Cardiac and Cerebral Diseases, the Second Affiliated Hospital of Guangdong Medical University, Zhanjiang, 524000, China

^3^ The Intensive Care Unit, Guangdong Key Laboratory of Age-Related Cardiac and Cerebral Diseases, Affiliated Hospital of Guangdong Medical University, Zhanjiang, 524000, China

^4^ Zhanjiang Key Laboratory of Organ Injury and Protection and Translational Medicine, Guangdong, 524000, China

^5^ The Intensive Care Unit, Jieyang Affiliated Hospital, Sun Yat-Sen University, Jeiyang, 52200, China

^6^ The Intensive Care Unit ,The Central Hospital of Wuhan, Tongji Medical College, Huazhong University of Science and Technology. Wuhan, 430000, China

^7^ The Intensive Care Unit, the Second Affiliated Hospital of Harbin Medical University, Harbin, China.

*Correspondence: cuilili@gdmu.edu.cn;


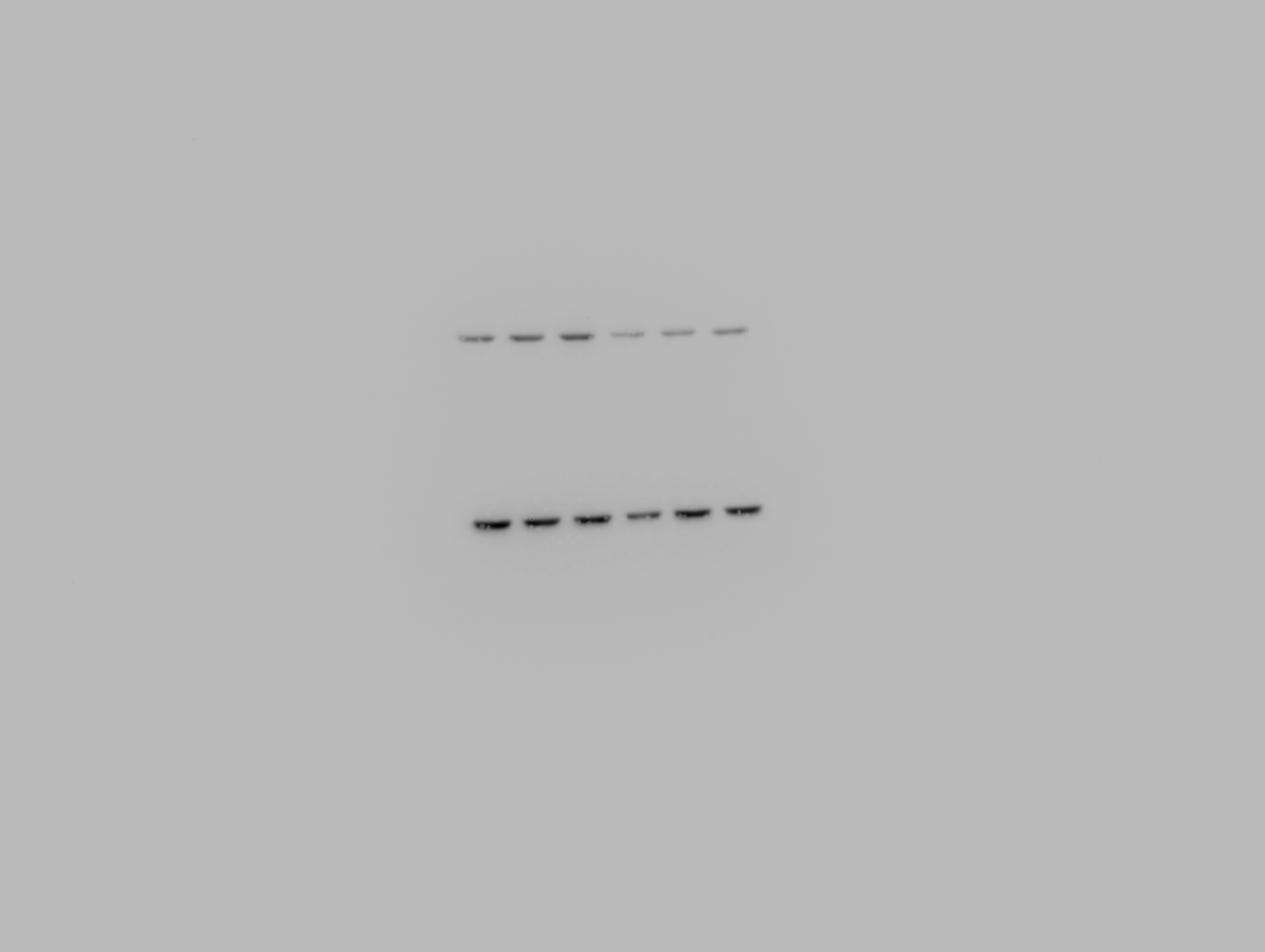


Supplementary Figure S1. The original, unprocessed versions of the effects of LPS on the expression of APOE in cultured raw264.7 cells for 24h was detected by western blot analysis.
